# Comparison of clinical outcome between laparoscopic and open right hemicolectomy: a nationwide study

**DOI:** 10.1186/s12957-015-0666-7

**Published:** 2015-08-15

**Authors:** Cha-Ze Lee, Li-Ting Kao, Herng-Ching Lin, Po-Li Wei

**Affiliations:** Division of Gastroenterology, Department of Internal Medicine, National Taiwan University Hospital, Taipei, Taiwan; Graduate Institute of Life Science, National Defense Medical Center, Taipei, Taiwan; School of Health Care Administration, Taipei Medical University, Taipei, Taiwan; Division of General Surgery, Department of Surgery, Taipei Medical University Hospital, Taipei, Taiwan; Department of Surgery, School of Medicine, College of Medicine, Taipei Medical University, 250 Wu-Hsing St., Taipei, 110 Taiwan; Cancer Center, Taipei Medical University Hospital, Taipei Medical University, Taipei, Taiwan

**Keywords:** Hemicolectomy, Laparoscopic, Outcome measures

## Abstract

**Background:**

This study aimed to compare clinical outcome between laparoscopic and open right hemicolectomy.

**Methods:**

The data were sourced from Taiwan’s National Health Insurance Research Database. This study included 14,320 and 1313 patients who underwent open and laparoscopic right hemicolectomies, respectively. The study outcome included “intensive care unit (ICU) admission,” “over 2 h of general anesthesia,” “use of mechanical ventilation,” “acute respiratory failure,” “in-hospital death,” and “hospitalization for pneumonia.” Separate conditional logistic regressions were performed for each clinical outcome.

**Results:**

The results showed that patients who underwent an open right hemicolectomy had significantly higher likelihood of ICU admission (31.4 vs. 13.4 %, *p* < 0.001), acute respiratory failure (3.6 vs. 0.8 %, *p* < 0.001), mechanical ventilation (12.8 vs. 4.1 %, *p* < 0.001), in-hospital death (3.7 vs. 0.9 %, *p* < 0.001), over 2 h of general anesthesia (4.6 vs. 1.2 %, *p* < 0.001), and hospitalization for pneumonia (5.8 vs. 3.1 %, *p* < 0.001) than patients who underwent a laparoscopic right hemicolectomy. Adjusted conditional logistic regression analyses revealed that patients who underwent an open right hemicolectomy were 2.96, 4.98, 3.41, 4.01, 3.44, and 1.78 times more likely to be admitted to the ICU, to have acute respiratory failure, the use of mechanical ventilation, in-hospital death, over 2 h of general anesthesia, and hospitalization for pneumonia, respectively, than patients who underwent a laparoscopic right hemicolectomy.

**Conclusions:**

Laparoscopic right hemicolectomy reduced risk of post-operative pulmonary complications.

## Background

Although the incidence of colorectal cancer decreased in some countries such as USA, UK, Australia, Japan, and Singapore [1], colorectal cancer is still one of the most common diagnosed malignancies in the world [2]. Since the first laparoscopic colon resection was reported in 1991 [3], the prevalence persisted low till 2004 because of technical difficulty and oncologic concern [4]. However, several multi-institutional prospective randomized trials including COST, COLOR, and CLASSIC studies demonstrated that laparoscopic colectomy for colon cancer provided better recovery with comparable oncologic results to open surgery [5–7]. Furthermore, for rectal cancer, COLOR II and COREAN trials demonstrated that laparoscopic rectal cancer surgery could be a safe procedure and it provided better post-operative recovery without compromise of short term oncological results [8–10].

However, according to our knowledge, no empirical study has attempted to explore clinical outcome for transverse colon cancers because of technical difficulty with lymph node dissection along middle colon artery. Some case series demonstrated that laparoscopic surgery for transverse colon cancer was technically feasible and safe for long-term oncologic outcomes. Conventional open surgery for transverse colon cancer needs upper abdominal incision. Upper abdominal incision frequently impaired post-operative pulmonary complications and resulted in the increase of morbidity [11, 12], mortality [13], hospital length of stay, and costs [12, 14]. A small incision placed around the umbilicus or low abdomen following laparoscopic right hemicolectomy for transverse colon cancers may be expected to reduce post-operative pulmonary complications then reduce length of hospital stay and costs. This study aimed to compare clinical outcome between laparoscopic and open right hemicolectomy through a nationwide database.

## Methods

### Database

The hospitalization data for this nationwide population-based study were sourced from Taiwan’s National Health Insurance Research Database (NHIRD). The NHIRD was published by the National Health Research Institute and was derived from the system of the Taiwan National Health Insurance (NHI), which was initiated in 1995. Taiwan’s NHI has a unique combination of characteristics: universal coverage, a single-payer payment system with the government as the sole insurer, comprehensive benefits, access to any medical institution of the patient’s choice, low out-of-pocket payment, and a wide variety of providers well distributed throughout the country. This dataset includes all medical claims data, such as medical expenditures, patients’ demographics, diagnostic codes, operation codes, et cetera from Taiwan’s NHI program. The NHI program was implemented as a means of financing healthcare for all Taiwanese citizens. As of 2007, 22.60 million of Taiwan’s 22.96 million population have been enrolled in the NHI program. The NHIRD provides an exclusive opportunity for the researchers to follow up on all the medical utilizations of all the enrollees under the Taiwan NHI since 1995. Many researchers have utilized the NHIRD to conduct and publish their studies in internationally peer-reviewed journals.

This study was exempted from full review by the Taipei Medical University Institutional Review Board (IRB) since the NHIRD consists of de-identified secondary data released to the public for research purposes.

### Study sample

We identified 15,906 transverse colon cancer patients who underwent a right hemicolectomy between January 2005 and December 2009 based on the ICD-9-CM procedure code 45.73 and 45.74 and ICD-9 disease code 153.0 and 153.1. We excluded 76 patents aged less than 18 years in order to limit our study sample to adult population. Moreover, we excluded patients who have been diagnosed with carcinomatosis (ICD-9-CM 199.0), or patients with peritonitis (ICD-9-CM 567.8) or colon perforation (ICD-9-CM 569.83) who would receive emergent operation (*n* = 197).

Of these 15,633 cases, we further identified those who underwent a laparoscopy by the additional ICD-9-CM procedure code 54.21. As a result, 14,320 and 1313 patients underwent open and laparoscopic right hemicolectomy, respectively, during the period between 2005 and 2009.

### Key variables of interest

All the variables used for this study were retrieved from inpatient claims. The primary study outcome were all binary variables, including “intensive care unit (ICU) admission,” “over 2 h of general anesthesia,” “use of mechanical ventilation,” “acute respiratory failure,” “in-hospital death,” and “hospitalization for pneumonia.” The independent variable of interest was whether or not a laparoscopy was used for right hemicolectomy.

In this study, we also take potential confounders including the characteristics of patients, surgeon, and hospital into consideration in the regression modeling. Patient characteristics consisted of age, gender, and severity of illness. Since no illness severity index is currently available in the NHIRD, we used the Charlson comorbidity index (CCI) to quantify pre-existing comorbidities as a means of adjusting for the higher potential mortality risks associated with 19 conditions (congestive heart failure, myocardial infarction, liver disease, cancer, dementia, etc.) with the score set at zero in the absence of comorbid conditions. In this study, a patient was stratified into five levels based on CCI score: 0, 1, 2, 3, 4, and ≥5. Surgeon characteristics included physician’s gender and age (as a surrogate for practice experience, <40, 40–49, and >49). Hospital characteristics included hospital teaching status and geographical location (Northern, Central, Southern, or Eastern Taiwan).

### Statistical analysis

The SAS statistical package (SAS System for Windows, Version 8.2) was used to perform the analyses in this study. We performed Pearson *χ*^2^ tests to examine the differences between patients who underwent a right hemicolectomy, in terms of characteristics of patient, surgeon, and hospital. Separate conditional logistic regressions (conditioned on hospitals and surgeon in order to partition out systematic hospital-specific and surgeon-specific variation) were carried out for each clinical outcome, in order to adjust for the characteristics of patient, surgeon, hospital, and potential clustering effects among patients treated by any given surgeon or hospital. A two-sided *p* value of less than, or equal to, 0.05 was considered to be statistically significant.

## Results

This study analyzed 14,320 who underwent an open right hemicolectomy and 1313 patients who underwent a laparoscopic right hemicolectomy. Overall, the mean age for the sampled patients was 64.0 years with a standard deviation of 14.9 years. Table 1 presents the distributions of characteristics of patient, surgeon, and hospital according to the use of laparoscopy. It shows that there was no statistically significant difference in the distribution of gender between patients who underwent an open right hemicolectomy and patients who underwent a laparoscopic right hemicolectomy. However, patients who underwent open right hemicolectomy were more likely to have a CCI score ≥5 than patients who underwent a laparoscopic right hemicolectomy (*p* < 0.001). In addition, there were significant differences in age group, hospital teaching status, hospital location, surgeon gender, and age between patients who underwent an open right hemicolectomy and patients who underwent a laparoscopic right hemicolectomy (all *p* < 0.001).

**Table 1:** Demographic characteristics of patients undergoing right hemicolectomy stratified by the presence of laparoscopy in Taiwan in the year 2005–2009 (*n* = 15,633)

Variable	Laparoscopic right hemicolectomy (*n* = 1313)		Open right hemicolectomy (*n* = 14,320)		*p* value
	Total no.	%	Total no.	%	
Patient characteristics					
Male	674	51.3	7550	52.7	0.334
Age					<0.001
18–24	14	1.1	91	0.6	
25–34	59	4.5	447	3.1	
35–44	119	9.1	1031	7.2	
45–54	224	17.1	2193	15.3	
55–64	295	22.5	2713	19.0	
>64	602	45.9	7845	54.8	
Charlson comorbidity index score					<0.001
0	320	24.4	1896	13.2	
1–2	65	5.0	631	4.4	
3	489	37.2	4582	32.0	
4	130	9.9	1586	11.1	
≥5	309	23.5	5625	39.3	
Hospital characteristics					
Hospital teaching status					<0.001
Yes	1281	97.6	13,339	93.2	
Hospital Location					<0.001
Northern	687	52.3	7011	49.0	
Central	340	25.9	2796	19.5	
Southern	224	17.1	4282	29.9	
Eastern	62	4.7	231	1.6	
Surgeon characteristics					
Male	1304	99.3	14,093	98.4	0.012
Age					<0.001
<40	365	27.8	3650	25.5	
40–49	767	58.4	6855	47.9	
>49	181	13.8	3811	26.6	

The distribution of adverse clinical outcomes between patients who underwent an open right hemicolectomy and patients who underwent a laparoscopic right hemicolectomy is shown in Table 2. Patients who underwent an open right hemicolectomy had significantly higher likelihood of ICU admission (31.4 vs. 13.4 %, *p* < 0.001), acute respiratory failure (3.6 vs. 0.8 %, *p* < 0.001), mechanical ventilation (12.8 vs. 4.1 %, *p* < 0.001), in-hospital death (3.7 vs. 0.9 %, *p* < 0.001), over 2 h of general anesthesia (4.6 vs. 1.2 %, *p* < 0.001), and hospitalization for pneumonia (5.8 vs. 3.1 %, *p* < 0.001) than patients who underwent a laparoscopic right hemicolectomy.

**Table 2:** Crude odds ratio for adverse clinical outcomes for right hemicolectomy patients with and without laparoscopy in Taiwan

Outcome	Total sample (*n* = 15,633)		Laparoscopic right hemicolectomy (*n* = 1313)		Open right hemicolectomy (*n* = 14,320)	
	No.	%	No.	%	No.	%
Intensive care unit admission						
Yes	4677	29.9	176	13.4	4501	31.4
Crude OR (95 % CI)	–		1.00		2.96* (2.52–3.48)	
Acute respiratory failure						
Yes	520	3.3	10	0.8	510	3.6
Crude OR (95 % CI)	–		1.00		4.81* (2.57–9.02)	
Mechanical ventilation						
Yes	1888	12.1	54	4.1	1834	12.8
Crude OR (95 % CI)	–		1.00		3.43* (2.60~4.52)	
In-hospital death						
Yes	546	3.5	12	0.9	534	3.7
Crude OR (95 % CI)	–		1.00		4.20* (2.36–7.46)	
Intratracheal intubation general anesthesia						
Over 2 h, each 30 min added						
Yes	670	4.3	16	1.2	654	4.6
Crude OR (95 % CI)	–		1.00		3.88* (2.36–6.39)	
Hospitalization for pneumonia						
Yes	869	5.6	40	3.1	829	5.8
Crude OR (95 % CI)			1.00		1.96* (1.42–2.70)	

*p < 0.001

Table 2 also shows the crude (OR) of ICU admission, acute respiratory failure, the use of mechanical ventilation, in-hospital death, over 2 h of general anesthesia, and hospitalization for pneumonia. Conditional logistic regression analyses (conditioned on hospitals and surgeon) revealed that patients who underwent an open right hemicolectomy were 2.96 (95 % CI = 2.52–3.48), 4.81 (95 % CI = 2.57–9.02), 3.43 (95 % CI = 2.60–4.52), 4.20 (95 % CI = 2.36–7.46), 3.88 (95 % CI = 2.36–6.39), and 1.96 (95 % CI = 1.42–2.70) times more likely to be admitted to the ICU, to have acute respiratory failure, the use mechanical ventilation, in-hospital death, over 2 h of general anesthesia, and hospitalization for pneumonia, respectively, than patients who underwent a laparoscopic right hemicolectomy.

Table 3 presents the independent variables for adverse clinical outcomes for right hemicolectomy patients with and without laparoscopy. Conditional logistic regression analyses revealed that, after adjusting for patients’ gender, age group, and CCI score, and surgeon’s age and gender, and hospital teaching status and geographic location, patients who underwent an open right hemicolectomy were 2.96 (95 % CI = 2.50–3.50), 4.98 (95 % CI = 2.64–9.37), 3.41 (95 % CI = 2.58–4.53), 4.01 (95 % CI = 2.25–7.16), and 3.44 (95 % CI = 2.08–5.69) times more likely to be admitted to the ICU, to have acute respiratory failure, the use of mechanical ventilation, in-hospital death, over 2 h of general anesthesia, and hospitalization for pneumonia, respectively, than patients who underwent a laparoscopic right hemicolectomy.

**Table 3:** Variables for adverse clinical outcomes for right hemicolectomy patients with and without laparoscopy in Taiwan

Variables	Intensive care unit admission	Acute respiratory failure	Mechanical ventilation
Open right hemicolectomy	2.96*** (2.50–3.50)	4.98*** (2.64–9.37)	3.41*** (2.58–4.53)
Patient characteristics			
Sex	1.03 (0.96–1.11)	1.13 (0.94–1.35)	1.11* (1.00–1.22)
Age	1.76*** (1.69–1.83)	1.90*** (1.69–2.14)	2.03*** (1.90–2.17)
Charlson comorbidity index score			
0			
1	1.65*** (1.35–2.02)	0.99 (0.67–1.48)	1.59*** (1.25–2.03)
2	4.66*** (3.02–7.18)	1.80 (0.98–3.32)	2.79*** (1.84–4.25)
3	0.60*** (0.53–0.68)	0.42*** (0.32–0.55)	0.47*** (0.40–0.56)
4	0.93 (0.80–1.08)	0.48*** (0.34–0.67)	0.77** (0.64–0.94)
≥5	0.82** (0.73–0.92)	0.43*** (0.33–0.56)	0.56*** (0.48–0.66)
Hospital characteristics			
Hospital teaching status	1.06 (0.92–1.23)	0.69* (0.51–0.95)	0.60*** (0.50–0.72)
Hospital location			
Northern			
Central	0.77*** (0.70–0.85)	1.89*** (1.52–2.36)	0.95 (0.83–1.09)
Southern	1.07 (0.99–1.16)	1.30* (1.05–1.61)	1.00 (0.89–1.13)
Eastern	1.44** (1.11–1.87)	2.18** (1.26–3.78)	1.07 (0.73–1.56)
Surgeon characteristics			
Sex	1.08 (0.80–1.45)	0.73 (0.41–1.30)	1.04 (0.72–1.52)
Age	0.75 (0.71–0.78)	0.68*** (0.59–0.77)	0.70*** (0.62–0.72)
Variables	In-hospital death	Intratracheal intubation general anesthesia	Hospitalization for pneumonia
Open right hemicolectomy	4.01*** (2.25–7.16)	3.44*** (2.08–5.69)	1.78*** (1.28–2.47)
Patient characteristics			
Sex	1.16 (0.97–1.38)	1.19* (1.02–1.39)	1.73*** (1.50–2.00)
Age	1.45*** (1.32–1.60)	1.66*** (1.51–1.83)	2.21*** (1.99–2.46)
Charlson comorbidity index score			
0			
1	1.60* (1.08–2.37)	1.65** (1.14–2.39)	1.74** (1.23–2.47)
2	1.45 (0.70–3.00)	1.67 (0.86–3.26)	1.10 (0.53–2.27)
3	0.47*** (0.35–0.63)	0.72* (0.55–0.94)	0.67** (0.52–0.87)
4	0.61** (0.43–0.87)	0.97 (0.72–1.32)	1.10 (0.83–1.45)
≥5	0.83 (0.64–1.09)	0.68** (0.52–0.88)	0.91 (0.72–1.12)
Hospital characteristics			
Hospital teaching status	0.59*** (0.44–0.79)	0.66** (0.50–0.86)	0.86 (0.66–1.13)
Hospital location			
Northern			
Central	0.82 (0.65–1.04)	1.10 (0.88–1.37)	1.39*** (1.16–1.66)
Southern	0.82 (0.67–1.01)	1.60*** (1.34–1.90)	1.15 (0.98–1.35)
Eastern	0.96 (0.50–1.83)	0.71 (0.33–1.52)	1.25 (0.75–2.08)
Surgeon characteristics			
Sex	0.45*** (0.28–0.71)	1.01 (0.54–1.88)	0.67 (0.42–1.08)
Age	0.69*** (0.61–0.78)	0.94 (0.84–1.05)	0.84*** (0.76–0.92)

*p < 0.05; **p < 0.01; ***p < 0.001

## Discussion

Several prospective randomized studies demonstrated that laparoscopic colectomy provided better post-operative recovery for patients with colon cancer, but patients with transverse colon cancer were excluded from these studies. Although some case series revealed laparoscopic colectomy can be safely performed for transverse colon cancers, a large scale study is still lacking. In this study, we demonstrated that as compared to patients after laparoscopic surgery for transverse colon cancers, patients after open surgery had higher ICU admission, incidence of acute respiratory failure, mechanical ventilation dependence, in-hospital mortality, intratracheal intubation general anesthesia, and hospitalization for pneumonia.

Pulmonary complications such as atelectasis and acute respiratory failure are the most common causes of morbidity and mortality in the post-operative period [15]. Surgical factors associated with pulmonary complications include type of surgery and extent of the surgical incision [16–19]. Conventional open surgery for transverse colon cancers often requires upper abdominal incision which is associated with several pulmonary complications. The incidence of pulmonary complications after upper abdominal surgical procedures was reported from 17~88 %, depending on the criteria for defining them [20]. Additionally, incisional pain and lying in the bed may contribute the shallow and monotonous breathing which will further induce development of pulmonary complications. Laparoscopic right hemicolectomy only requires one 5~6 cm small incision to retrieve surgical specimens, which can be placed at the low abdomen or peri-umbilical area. Incisional pain is less severe with laparoscopic right hemicolectomy because of smaller incisional wound, and laparoscopic right hemicolectomy does not need an upper abdominal incision to retrieve surgical specimens. With these two factors, laparoscopic right hemicolectomy may decrease post-operative pulmonary complications.

There are no reliable evidence-based predictors that can identify post-abdominal surgery patients who will require prolonged mechanical ventilation. In this study, we demonstrate that conventional open surgery increases likelihood of post-operative mechanical ventilation dependence among patients with transverse colon cancer. Mechanical ventilation dependence can cause several systemic adverse effects including lower cardiac output [21, 22], increased risk of gastrointestinal complications [23–25], inducing inflammation [26], and systemic muscular weakness [27]. Patients of mechanical ventilation dependence often need ICU admission which will further increase hospital cost. In comparison with laparoscopic surgery, conventional open surgery increases the risk of post-operative mechanical ventilation dependence.

Post-operative mortality is the most serious adverse event after abdominal surgery. Fortunately, the rates of post-operative mortalities are low [28]. Emergent operation, pre-operative morbidities, and high American society of anesthesiologists (ASA) score are associated with increased risk of post-operative mortality [29–32]. Management of acute respiratory failure in patients after abdominal surgery admitted to the ICU requires endotracheal intubation and mechanical ventilation. Furthermore, acute respiratory failure with the need of endotracheal intubation and mechanical ventilation are independent predictors of hospital mortality [33]. However, role of operative methods on mortality after abdominal surgery is still controversial. In this study, we demonstrated that laparoscopic colectomy was associated with lower risk of mortality after operation for transverse colon cancer. Lower risk of respiratory failure, mechanical ventilation dependence, and ICU admission are the advantages of laparoscopic right hemicolectomy which reduce the risk of post-operative mortality.

The main strength of this study is the usage of a nationwide population-based dataset from Taiwan which launched a universal NHI program at the end of 1995, and NHI program covered 98 % of Taiwan’s population. This study therefore excluded the influence of insurance status from our analysis which was demonstrated as an important factor influencing care of patients with colon cancer [34]. However, there are some limitations in this study. First, cancer stage, anastomotic leakage, and BMI cannot be obtained in this data set, which may influence the post-operative course. Secondly, pathologic results such as tumor size, lymph node retrieval number, and surgical margin are important factors to evaluate the feasibility of a new surgical method. In this data set, we cannot access those factors. Third, the open operation might be performed more frequently in patients with an acute disease. The dataset used in this study did not allow us to determine the severity or acuteness of disease which might confound the results.

## Conclusions

In conclusion, this study found that laparoscopic right hemicolectomy was associated with the reduced risk of post-operative pulmonary complications, ICU admission, and in-hospital death through a nationwide database in this study. Further epidemiological studies in other regions or countries are suggested to confirm the findings of present studies.
